# Ways to improve the methodology of meta-analysis in sports and exercise medicine: what do we often see in peer review?

**DOI:** 10.1136/bmjsem-2024-002256

**Published:** 2024-09-28

**Authors:** Patrick J Owen, Ishtiaq Ahmed, Aamir Raoof Memon, Nash Anderson, Evert Verhagen, Niamh L Mundell, Daniel L Belavy

**Affiliations:** 1 Eastern Health Emergency Medicine Program, Melbourne, Victoria, Australia; 2 Eastern Health Clinical School, Monash University, Melbourne, Victoria, Australia; 3 Pain in Motion Research Group (PAIN), Department of Physiotherapy, Human Physiology and Anatomy, Faculty of Physical Education & Physiotherapy, Vrije Universiteit Brussel, Brussels, Belgium; 4 Institute for Health and Sport, Victoria University, Melbourne, Victoria, Australia; 5 Tuggeranong Chiropractic Centre, Canberra, Australian Capital Territory, Australia; 6 Canberra City Chiro, Canberra, Australian Capital Territory, Australia; 7 Amsterdam Collaboration on Health & Safety in Sports, Department of Public and Occupational Health, Amsterdam Movement Sciences, Amsterdam UMC, Vrije Universiteit Amsterdam, Amsterdam, Netherlands; 8 Institute for Physical Activity and Nutrition, School of Exercise and Nutrition Sciences, Deakin University, Geelong, Victoria, Australia; 9 Hochschule für Gesundheit, Gesundheitscampus 6-8, Department of Allied Health Sciences, Bochum, Germany

**Keywords:** Meta-analysis, Sports, Exercise, Medicine

 Systematic reviews with meta-analyses of randomised controlled trials are often considered the highest form of scientific evidence. Meta-analyses are an indispensable tool offering a statistically powerful method to pool effect estimates from multiple studies. However, the methodology of meta-analysis is often fraught with complexities and challenges that can markedly impact conclusions and generalisability. We recently highlighted issues commonly observed as reviewers and editors of systematic reviews in sports and exercise medicine,^1^ yet methodological issues of meta-analysis warrant further discussion. Issues include, yet are not limited to, choice of statistical model, challenges posed by data paucity, evaluating certainty of evidence (eg, inconsistency and heterogeneity), and how findings are communicated. This editorial delves into these critical aspects, highlighting common pitfalls and offering guidance on best practices to enhance the use of meta-analyses in sports and exercise medicine.

## Statistical models for meta-analysis

Two widely used statistical models for meta-analysis are the fixed and random effects model. Researchers commonly use the concept of heterogeneity to determine whether a random or fixed-effect model is suitable for their analysis.^2^ However, selecting the correct statistical model should be based on the research question rather than heterogeneity. Random-effect models will typically be a better choice than fixed-effect models for the kinds of research questions assessed in sports and exercise medicine, such as comparing the effects of two interventions.^2^

## Meta-analysis with fewer than five studies

Data paucity is a common limitation when synthesising evidence in sports and exercise medicine. Unfortunately, authors may unknowingly exacerbate this limitation by overlooking the influence of data paucity when conducting meta-analysis. For example, six meta-analyses published in 2020 examined the same three studies comparing arthroscopic hip surgery to physical therapy among patients with femoroacetabular impingement syndrome, yet none employed appropriate meta-analytical methods to account for data paucity.^3^ When faced with less than five studies in a meta-analysis, we recommend adopting a random-effect model with the Hartung-Knapp-Sidik-Jonkman adjustment for a conservative estimate. Alternatively, it may be wise to use this as the default method, with an automatic adjustment for those rare but possible cases where tau=0.

## Unit of analysis error

When conducting a meta-analysis, authors may encounter a study with more than two arms (multiarm). In such cases, if one intervention arm is irrelevant, the data from that arm may be omitted from the analysis, and the study can be treated as a standard two-arm trial. However, if both arms are relevant, estimating the effect between both intervention arms and the same control group leads to double counting control group participants. This creates an error in the unit of analysis, where the meta-analysis fails to address the correlation between the estimated intervention effects from multiple comparisons. To address this issue, review authors can pool the two intervention groups into one group using the formula below or use other approaches recommended in the Cochrane Handbook.^4^,^6^

$$Sample\;size=N_{1}+N_{2}$$

$$Mean=\frac{N_{1}M_{1}+N_{2}M_{2}}{N_{1}+N_{2}}$$

$$Standard\;Deviation=\sqrt{\frac{\left(N_{1}-1\right)SD_{1}^{2}+\left(N_{2}-1\right)SD_{2}^{2}+\frac{N_{1}N_{2}}{N_{1}+N_{2}}+\left(M_{1}^{2}+M_{2}^{2}-2M_{1}M_{2}\right)}{N_{1}+N_{2}-1}}$$

## Certainty of Evidence using the Grading of Recommendations Assessment, Development and Evaluation approach

Grading of Recommendations Assessment, Development and Evaluation (GRADE), the most widely used tool for assessing evidence certainty, evaluates five domains (ie, risk of bias, indirectness, inconsistency, imprecision and publication bias) and classifies the certainty of evidence (for each outcome studied) as very low, low, moderate or high.^7^

### Risk of bias

Bias arises when the findings of a study are misleading due to inherent limitations in study design or conduct.^8^ It is important to note that the risk of bias may vary across outcomes. For example, loss to follow-up may be less of a limitation for all-cause mortality compared with health-related quality of life. Another point to consider is ‘follow-up’, whereby the risk of bias may differ at ‘short-term follow-up <3 months’ vs ‘long-term follow-up >12 months’ for reasons such as attrition. Despite this, systematic reviews often assess the risk of bias by studies across outcomes rather than by outcomes across studies. Review authors are advised against taking an approach that averages across studies when downgrading the evidence due to the risk of bias. Instead, authors should assess the risk of bias for individual outcomes across studies and adhere to the GRADE guidelines.^8^ Where resources are limited, authors should define the outcome and follow-up period used for evaluating the risk of bias a priori and consider how this may limit the overall interpretation of analyses.

### Indirectness

Confidence in effect estimates may decrease when the evidence does not come from research directly relevant to the population, intervention, comparator or outcomes of interest being considered in a systematic review. For example, in a review focusing on a physical activity intervention for the secondary prevention of coronary heart disease, most identified studies were instead conducted in individuals with type 2 diabetes. Consequently, the evidence may be considered indirect due to the predominant inclusion of people with diabetes.^9^ Review authors should follow GRADE guidelines when downgrading the evidence due to indirectness.^10^

### Inconsistency (heterogeneity)

Heterogeneity in a meta-analysis is often a misunderstood concept that has been discussed in depth elsewhere.^2^ In brief, heterogeneity refers to the distribution of true effect estimates across the studies in a meta-analysis and underscores the inconsistency criteria of the GRADE approach.^11^ When evaluating inconsistency (heterogeneity), authors often solely use numerical cut-offs based on the I^2^ statistic, such as those proposed by the Cochrane Handbook,^12^ which is not considered best practice.^2^ Rather, authors should first attempt to explain observed heterogeneity via methods such as meta-regression. Where heterogeneity remains unexplained, authors should then consider the Confidence IN Network Meta-Analysis approach that examines the 95% CI and 95% prediction interval (95%PI) with regard to thresholds of a defined region of equivalence.^13^ For example, a method adopted by a recent meta-analysis examining the effects of exercise training on preventing neck pain defined OR≤0.78 as the beneficial threshold and OR≥1.05 as the harmful threshold.^14^ Estimates were downgraded one level if either: (a) 95% CI crossed no threshold and 95% PI crossed one threshold or (b) 95% CI crossed one threshold and 95% PI crossed two thresholds. Estimates were downgraded two levels if 95% CI crossed no thresholds and 95% PI crossed two thresholds. Of note, when less than five studies are included in a meta-analysis, the tau statistic can assist in defining thresholds.^15 16^

### Imprecision

Imprecision can be assessed based on the width of CIs, optimal volume of information and assumed range of effects that may influence a recommendation. A narrow interval (eg, 0.60–0.70) indicates precise knowledge of the effect size. A wider interval (eg, 0.40–0.85) suggests greater uncertainty, but decisions about intervention utility may still be possible. Very wide intervals (eg, −0.15 to 1.20) signify limited knowledge, requiring additional information for a more certain conclusion. To rate imprecision, authors need to establish thresholds for outcomes corresponding to trivial or no, small, moderate or large effects and then downgrade the evidence by up to three levels based on serious, very serious and extremely serious concerns. For example, Cohen’s d effect sizes 0.2, 0.5 and 0.8 can be selected as thresholds.^17^ The authors can downgrade the estimate by two levels if the 95% CI crosses two thresholds (0.50 to 0.80). Review authors are recommended to predefine the threshold following GRADE guidelines and then downgrade the evidence due to imprecision.^17^

## Communication of findings

Most authors confuse ‘no evidence of an effect’ with ‘evidence of no effect’. When interpreting results, we advise authors against relying on binary terminology, such as ‘statistically significant’, ‘non-significant’ or ‘not statistically significant’. Instead, authors should communicate the meta-analysis findings based on the effect estimate and the certainty of the evidence. For example, we will consider the following estimate (Hedges’ g (95% CI)) comparing intervention to control for an arbitrary outcome: 0.40 (−0.05, 0.50), p>0.05, GRADE: low. The authors should not conclude that ‘the intervention has no effect’ based solely on the p value. Instead, because the 95% CI represents a range of values compatible with the true effect given these data, a better interpretation is that ‘the intervention may have a positive effect, yet may also have a negative effect’. The low certainty evidence per GRADE signifies additional data is likely required for a more certain conclusion and should be interpreted as ‘limited confidence in the effect estimate’ and that when additional data is considered ‘the true effect may be substantially different from the estimate’. We recommend that authors follow the Cochrane Handbook and/or GRADE guidelines to communicate the meta-analysis findings effectively.^18^

## Conclusion

The rigour and reliability of a meta-analysis is deeply rooted in robust methodology. This editorial highlighted several crucial aspects that authors could consider to enhance the quality of meta-analyses in sports and exercise medicine. Key among these are a nuanced understanding of heterogeneity and the GRADE model, the careful selection of statistical models, the mindful handling of analyses involving a small number of studies, avoiding unit of analysis error and the guidance on correctly communicating the findings of meta-analysis. By addressing these aspects, researchers can navigate the complexities inherent in meta-analytical methods, producing statistically sound findings. Researchers’ methodological diligence strengthens the evidence base in sports and exercise medicine, guiding future research and clinical practice towards more effective and evidence-based outcomes.
